# Decreases in Smoking-Related Cancer Mortality Rates Are Associated with Birth Cohort Effects in Korean Men

**DOI:** 10.3390/ijerph13121208

**Published:** 2016-12-05

**Authors:** Yon Ho Jee, Aesun Shin, Jong-Keun Lee, Chang-Mo Oh

**Affiliations:** 1 MRC Population Health Research Unit, Clinical Trial Service Unit and Epidemiological Studies Unit, Nuffield Department of Population Health, University of Oxford, Oxford OX3 7LF, UK; minniejee93@gmail.com; 2 Department of Preventive Medicine, Seoul National University College of Medicine, Seoul 03080, Korea; shinaesun@snu.ac.kr; 3 Cancer Research Institute, Seoul National University, Seoul 03080, Korea; 4 Radiation Epidemiology Team, Radiation Health Institute, Korea Hydro & Nuclear Power Co., Ltd., Seongnam 01450, Korea; jongkeun@khnp.co.kr; 5 Cancer Registration and Statistic Branch, National Cancer Control Institute, National Cancer Center, Goyang 10408, Korea

**Keywords:** smoking, cancer, mortality, trends, birth cohort, Korea

## Abstract

*Background:* This study aimed to examine trends in smoking-related cancer mortality rates and to investigate the effect birth cohort on smoking-related cancer mortality in Korean men. *Methods:* The number of smoking-related cancer deaths and corresponding population numbers were obtained from Statistics Korea for the period 1984–2013. Joinpoint regression analysis was used to detect changes in trends in age-standardized mortality rates. Birth-cohort specific mortality rates were illustrated by 5 year age groups. *Results:* The age-standardized mortality rates for oropharyngeal decreased from 2003 to 2013 (annual percent change (APC): −3.1 (95% CI, −4.6 to −1.6)) and lung cancers decreased from 2002 to 2013 (APC −2.4 (95% CI −2.7 to −2.2)). The mortality rates for esophageal declined from 1994 to 2002 (APC −2.5 (95% CI −4.1 to −0.8)) and from 2002 to 2013 (APC −5.2 (95% CI −5.7 to −4.7)) and laryngeal cancer declined from 1995 to 2013 (average annual percent change (AAPC): −3.3 (95% CI −4.7 to −1.8)). By the age group, the trends for the smoking-related cancer mortality except for oropharyngeal cancer have changed earlier to decrease in the younger age group. The birth-cohort specific mortality rates and age-period-cohort analysis consistently showed that all birth cohorts born after 1930 showed reduced mortality of smoking-related cancers. *Conclusions:* In Korean men, smoking-related cancer mortality rates have decreased. Our findings also indicate that current decreases in smoking-related cancer mortality rates have mainly been due to a decrease in the birth cohort effect, which suggest that decrease in smoking rates.

## 1. Introduction

Cigarette smoking is the most important health problem worldwide, accounting for an estimated six million deaths annually—both from cancer and other diseases [1]. In Korea, tobacco consumption increased from 1945—the year the Korean War ended—reaching a rate of 79.3% in men and 12.6% in women in the 1980s [2] before starting to decline. By 2015, smoking prevalence rates had decreased to 41.4% in men and 5.7% in women [3,4]. Cigarette smoking is recognized as a major risk factor for lung, oropharyngeal, esophageal, and laryngeal cancer and induce the misreplication of DNA and increased mutational signature [5]. The estimated attributable fraction due to smoking for death from these cancers is more than 50% [6]. Given the decrease in smoking rates since the mid-1980s, mortality rates for smoking-related cancers are likely to decline. However, few studies have examined this hypothesized trend in Korea.

Conversely, the disease burden from smoking-related cancers in Korean women was estimated to have increased from 351.8 disability-adjusted life years (DALY) in 2001 to 732.2 DALYs in 2008. Korean men have followed a similar pattern, with a DALY of 1930.1 in 2001 increasing to 2038.9 in 2008 [7,8]. In addition, although smoking rates have decreased since the 1980s, the incidence or mortality from cancer reflects *past* smoking rates—9–40 years prior to these outcomes occurring [9]. Therefore, the smoking effects on the cancer mortality rates are complicated. However, to our knowledge, there is no direct method to evaluate the attributable fraction for external effects on the changing disease trends in Korea, because it is difficult to obtain data on smoking rates prior to 1980.

Age-period-cohort modeling enables the separation of the effects of age, period, and cohort. Additionally, it allows description of their simultaneous effects on disease trends [10]. The cohort effect reflects how the unequal population level environmental exposure affects age groups differentially [11,12]. In cigarette smoking, it reflects the different smoking exposure of each birth cohort [13]. Therefore, it would be helpful to investigate the birth cohort effects on assessing the impact that reduced smoking rate has on the changing trends in smoking-related cancer mortality.

The aim of this study was to examine the secular trends in smoking-related cancer mortality rate and to investigate the birth cohort effect on smoking-related cancer mortality in Korean men.

## 2. Materials and Methods

### 2.1. Data Sources

We restricted our study to Korean men only because men have much higher smoking rates compared to women. Indeed, smoking rate in women is relatively low and stable compared to men in Korea [4]. According to the Tobacco Atlas published by the World Health Organization (WHO), the current smoking rate in Korean women was estimated as 5.9% in 2013 and the estimated number of current smokers among Korean women was 1,256,200 people [14].

We thus postulated that trends in cancer mortality in men would better reflect the effects of smoking. We defined smoking-related cancer as oropharyngeal, esophageal, laryngeal, and lung cancer [5,6,15]. The annual number of deaths from these cancers and the corresponding mid-year population counts from 1984 to 2013 were extracted from Statistics Korea data (Available from: http://kosis.kr/) [4]. Oropharyngeal, esophageal, laryngeal and lung cancer were defined as “C00-C14”, “C15”, “C32”, and “C34”, respectively, according to the 10th revision of the International Classification of Diseases (ICD-10) [16].

To examine the time trends in age-standardized mortality rates for oropharyngeal, esophageal, laryngeal and lung cancer, we conducted Joinpoint regression analysis for mortality rates of oropharyngeal, esophageal, laryngeal and lung cancer.

After estimating the time trends in age-standardized mortality rates, study participants were restricted to men aged ≥40 years old, because the mortality rate among men aged <40 years old was very low. To investigate the birth cohort effects on smoking-related cancer mortality, the mortality data were divided into six periods of five years each (1984–1988 to 2009–2013) and nine age groups of five years each (40–44 to ≥80 years). The consecutive fourteen birth cohort groups were calculated by subtracting age from the period (birth cohort = period − age). Ethics approval for the research protocol was granted by the institutional review board (IRB) of the National Cancer Center (IRB No.: NCC2015-0250, Goyang, Korea).

### 2.2. Statistical Analysis

Age-standardized mortality rates (ASMRs) for oropharyngeal, esophageal, laryngeal, and lung cancer were calculated by using Segi’s World Standard population as the standard population. Joinpoint regression modelling was used to test trends in ASMRs for oropharyngeal, esophageal, laryngeal and lung cancers and to establish significant changes over time to fit a better multi-segmented model compared with a simple linear model [17]. The trends in rates were summarized as annual percentage changes (APCs).

To evaluate the birth cohort effect on smoking-related cancer mortality, age was categorized into five-year age groups (from 40–44 years to ≥80 years of age). The birth cohort specific cancer mortality rate were stratified by age group to evaluate the cohort effects. To evaluate birth cohort effects after adjusting for age and period effects, a log-linear model using the intrinsic estimator (IE) method was performed, based on the assumption that the number of deaths in each age group followed a Poisson distribution [18,19]. The equation for log-linear regression model for age-period-cohort analysis is described as follows:

$$\text{log}\left({\text{number of cancer deaths}}_{ij}\right)=\text{log}\left({\text{corresponding population}}_{ij}\right)+Age_{i}+Period_{j}+Cohort_{k}+\varepsilon_{ij}$$

The final model was selected by goodness of fit test using Akaike information criterion (AIC) and likelihood ratio test (Supplementary Materials Table S1). *p*-values less than 0.05 were considered statistically significant. The statistical analysis was performed using SAS software version 9.3 (SAS Institute Inc., Cary, NC, USA, 2012), Stata Statistical Software Release 12 (StataCorp LP, College Station, TX, USA, 2011) and Joinpoint Regression Program software version 4.1.1 (Statistical Methodology and Applications Branch, Surveillance Research Program, National Cancer Institute: Bethesda, MD, USA).

## 3. Results

### 3.1. Number and Proportion of Deaths from Smoking Related Cancer

Between 1984 and 2013, the total number of deaths for all cancer sites combined among Korean men was 1,024,976 (Table 1). Of these, 286,914 deaths (28.0%) were from smoking-related cancers: 14,679 (1.4%) from oropharyngeal; 34,782 (3.3%) from esophageal; 15,356 (1.5%) from laryngeal; and 222,097 (22.7%) from lung cancer. Between 2004 and 2013, the number of deaths from smoking-related cancers was about 2.7 times greater than in the decade spanning 1984 to 1993.

### 3.2. Trends in Age-Standardized Mortality Rates for Smoking Related Cancer

**Figure 1:**
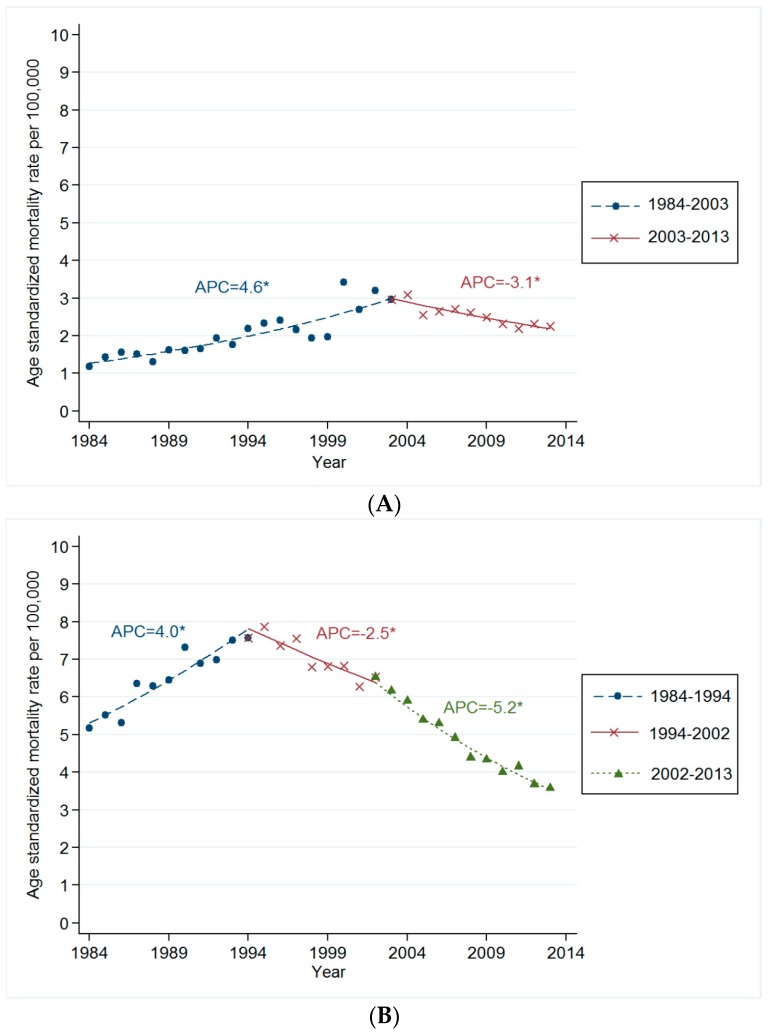

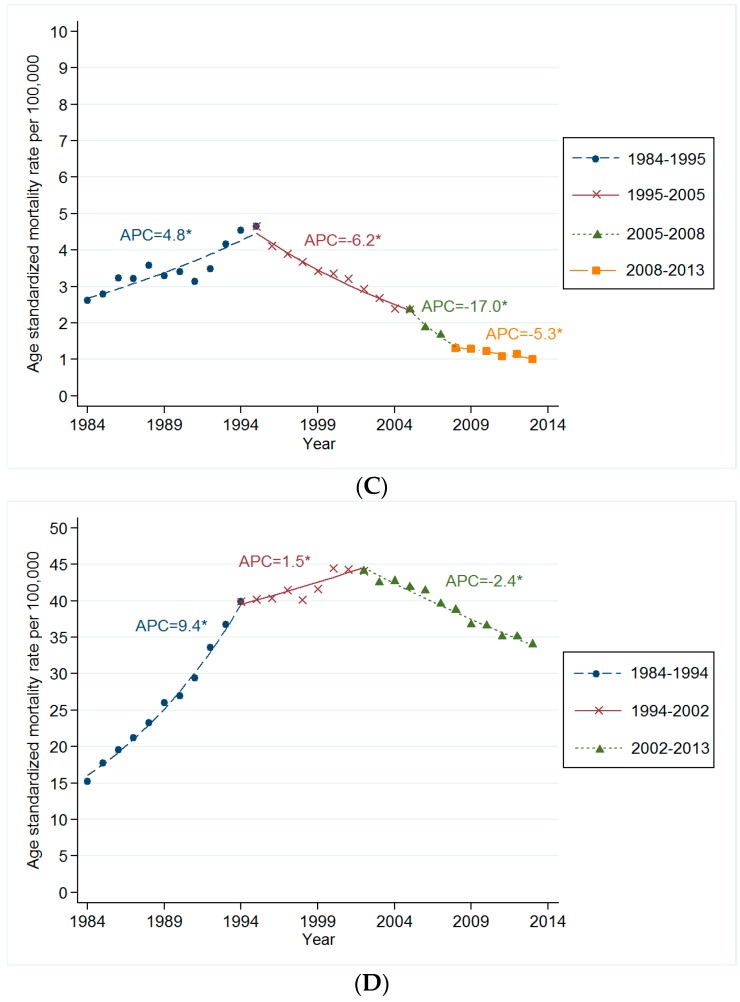
(**A**) Trends in age-standardized mortality rate for oropharyngeal cancer in Korean men. Footnotes: The age-standardized mortality rates are presented as number of oropharyngeal cancer per 1,000,000 people using Segi’s world standard population as standard population. Joinpoint regression analysis was used to determine whether there were significant changes in trends in age-standardized mortality rates for the period between 1984 and 2013; (**B**) Trends in age-standardized mortality rate for esophageal cancer in Korean men. Footnotes: The age-standardized mortality rates are presented as number of esophageal cancer per 1,000,000 people using Segi’s world standard population as standard population. Joinpoint regression analysis was used to determine whether there were significant changes in trends in age-standardized mortality rates for the period between 1984 and 2013; (**C**) Trends in age-standardized mortality rate for laryngeal cancer in Korean men. Footnotes: The age-standardized mortality rates are presented as number of laryngeal cancer per 1,000,000 people using Segi’s world standard population as standard population. Joinpoint regression analysis was used to determine whether there were significant changes in trends in age-standardized mortality rates for the period between 1984 and 2013; (**D**) Trends in age-standardized mortality rate for lung cancer in Korean men. Footnotes: The age-standardized mortality rates are presented as number of lung cancer per 1,000,000 people using Segi’s world standard population as standard population. Joinpoint regression analysis was used to determine whether there were significant changes in trends. * *p* < 0.05.

Recent trends show that the ASMRs from these smoking-related cancers have begun to decrease. The mortality rate for oropharyngeal cancer has decreased from 2003 to 2013 (APC: −3.1 (95% CI, −4.6 to −1.6)), as shown in Figure 1A. The esophageal cancer mortality rate has shown a decreasing trend from 1994 to 2002, preceding the mortality rate decline observed for oropharyngeal cancer (APC: −2.5 (95% CI, −4.1 to −0.8)). Furthermore, the esophageal cancer mortality rate decreased more rapidly from 2002 to 2013 (APC: −5.2 (95% CI, −5.7 to −4.7)), as shown in Figure 1B. Figure 1C shows how the laryngeal cancer mortality rate has declined since 1995 (APC: −6.2 (95% CI, −6.2 to −7.7) from 1995 to 2005, APC: −17.0 (95% CI, −27.1 to −5.7) from 2005 to 2008; APC: −5.3 (95% CI, −7.4 to −3.1) from 2008 to 2013).

The annual percentage increase of lung cancer has slowed, from an increase of 9.4% (95% CI, 8.7 to 10.2) between 1984 and 1994, to an increase of 1.5% (95% CI, 0.6 to 2.4) between 1994 and 2002. After 2002, the mortality rate of lung cancer has reduced (APC −2.4; 95% CI −2.7 to −2.2), shown in Figure 1D.

### 3.3. Changes in Mortality Rates for Smoking Related Cancer by Time Period and Birth Cohort

**Figure 2:**
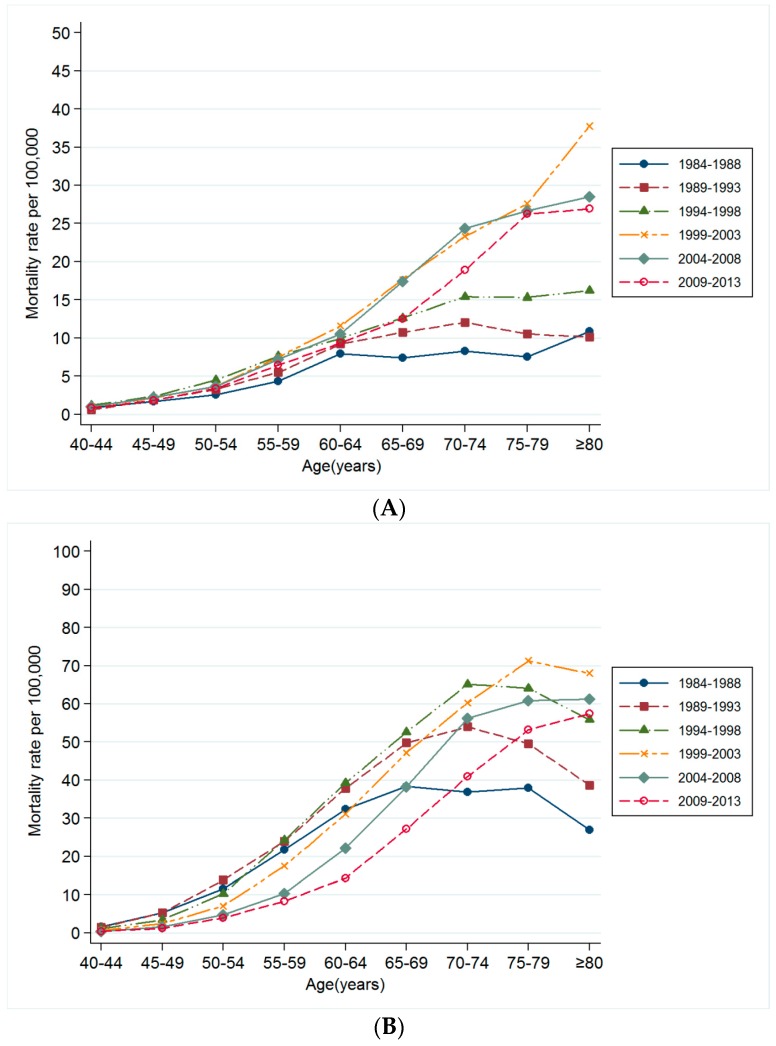

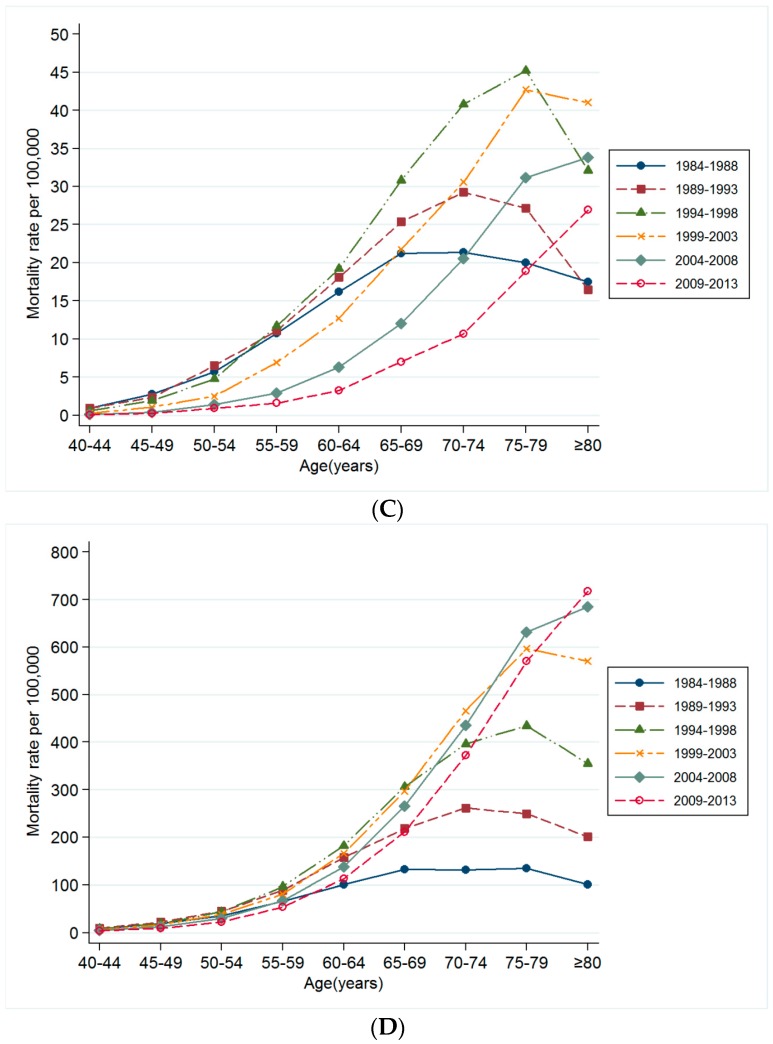
(**A**) Change in age-specific mortality rate for oropharyngeal cancer by time period; (**B**) Change in age-specific mortality rate for esophageal cancer by time period; (**C**) Change in age-specific mortality rate for laryngeal cancer by time period; (**D**) Change in age-specific mortality rate for lung cancer by time period.

According to the age group, the mortality rates for oropharyngeal cancer, esophageal cancer and laryngeal cancer showed the highest mortality rate for the period of 1999–2003, and then leveled off for the period of 2004–2008 and 2009–2013 among ≥80 years old age group (Figure 2A–C). On the other hands, the mortality rate for the lung cancer was highest for the period of 2009–2013 among ≥80 years old age group, but the mortality rate for the lung cancer for the period of 2009–2013 was lower than for the period of 2004–2008 among people aged 40–79 (Figure 2D).

**Figure 3:**
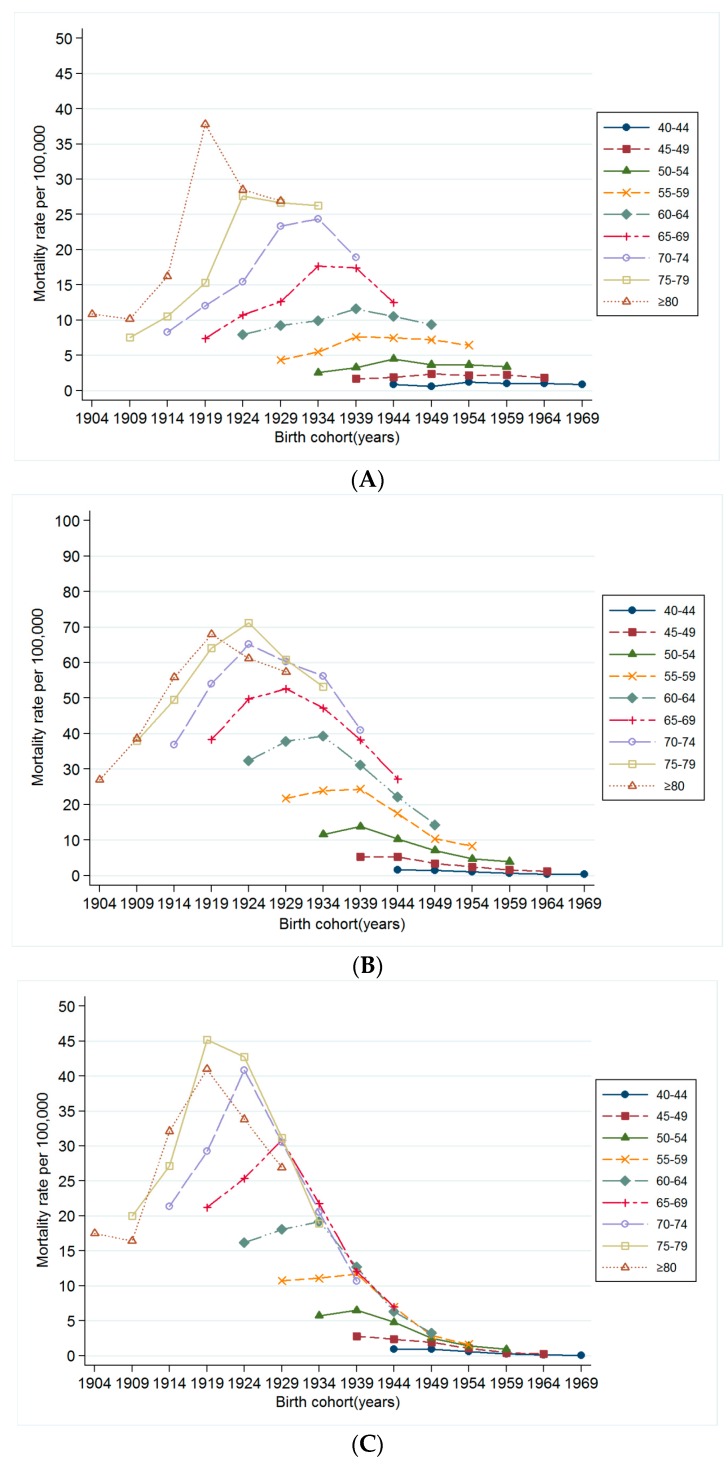

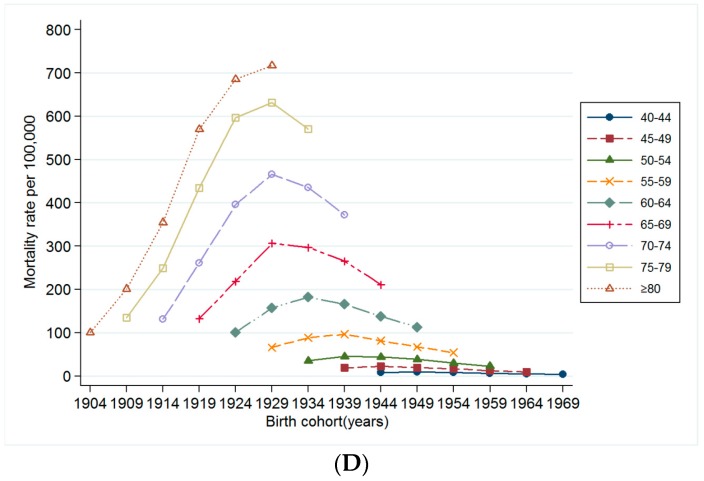
(**A**) Change in birth cohort specific mortality rate for oropharyngeal cancer by age group; (**B**) Change in birth cohort specific mortality rate for esophageal cancer by age group; (**C**) Change in birth cohort specific mortality rate for laryngeal cancer by age group; (**D**) Change in birth cohort specific mortality rate for lung cancer by age group.

**Figure 4:**
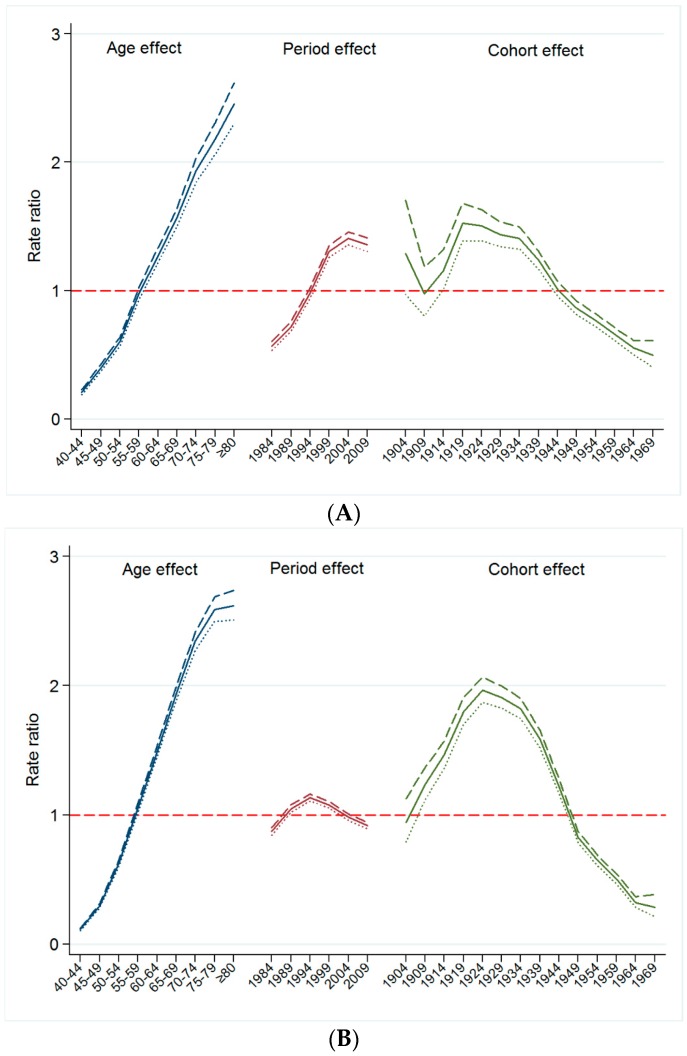

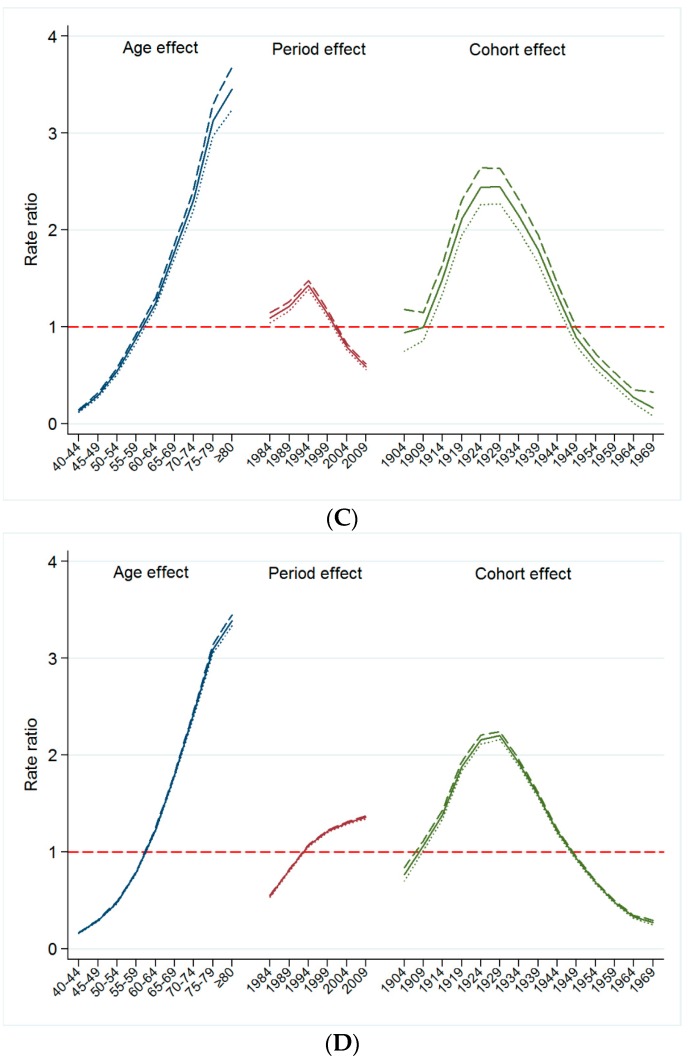
(**A**) Birth cohort effects on the oropharyngeal cancer mortality rate. Footnotes: The cohort effects on the oropharyngeal cancer mortality are estimated by a log-linear model using the intrinsic estimator (IE) method and expressed as risk ratios. The cohort effects and their 95% confidence intervals on the oropharyngeal cancer mortality represent as solid lines and dashed lines, respectively; (**B**) Birth cohort effects on the esophageal cancer mortality rate in Korean men. Footnotes: The cohort effects on the esophageal cancer mortality are estimated by a log-linear model using the intrinsic estimator (IE) method and expressed as risk ratios. The cohort effects and their 95% confidence intervals on the esophageal cancer mortality represent as solid lines and dashed lines, respectively; (**C**) Birth cohort effects on the laryngeal cancer mortality rate in Korean men. Footnotes: The cohort effects on the laryngeal cancer mortality are estimated by a log-linear model using the intrinsic estimator (IE) method and expressed as risk ratios. The cohort effects and their 95% confidence intervals on the laryngeal cancer mortality represent as solid lines and dashed lines, respectively; (**D**) Birth cohort effects on the lung cancer mortality rate in Korean men. Footnotes: The cohort effects on the lung cancer mortality are estimated by a log-linear model using the intrinsic estimator (IE) method and expressed as risk ratios. The cohort effects and their 95% confidence intervals on the lung cancer mortality represent as solid lines and dashed lines, respectively.

Figure 3 presents the change in mortality rates of smoking-related cancers by birth cohort year within each corresponding age group, Although it was difficult to detect changes in the mortality rate in the younger age groups, decreasing trends were shown in the older groups (aged ≥60 years). In the age-period-cohort model, the risk ratio for smoking-related cancer mortality has decreased among those born after 1929 after adjusting for age and period effects (Figure 4). These decreasing birth cohort effects for cancer mortality were observed for all smoking-related cancers (Figure 4A–D).

## 4. Discussion

Our study showed that mortality from various smoking-related cancers has decreased since 1995 or 2003. Although there were cancer-specific differences in when mortality rate (of the four smoking-related cancers) decreases started, the pattern was very similar. The age-standardized mortality rates for smoking-related cancers have decreased later as age increases. This study also shows that birth cohorts born in the 1920–1930s had the highest smoking-related cancer mortality rate. The birth cohort effects subsequently leveled off, and then began to decrease in younger generations.

In our study, the decrease in the smoking-related cancer mortality rate began at a similar point in time for oropharyngeal and lung cancer, and for esophageal and laryngeal cancer. Although well known that these four cancer types are especially related to smoking, the different time points at which their mortality rates began to decrease were likely due to the different period effects, reflecting advances in treatment. Indeed, the starting points and patterns of mortality decrease given birth cohort effects for these smoking-related cancers were similar. The decreasing birth cohort pattern for lung cancer mortality parallels the age-period-cohort analyses in Spain [20], the United States of America [21], China [22] and Japan [23]. These studies show the birth cohorts born in the 1950–1960s had the highest mortality or incidence rate of lung cancer, which then leveled off, and began to decrease in the younger generations. Conventional age-period-cohort model is suffered from identification problem, because age, period and cohort variables are perfectly collinear. Therefore, we implemented the intrinsic estimator method to solve this problem [18]. In Japan, age-standardized mortality rate for lung cancer started to decrease since 1996 [24]. For this Japanese study, lung cancer mortality has decreased among those born in the late 1930s. However, this finding was not adjusted for age effect and period effect. Veteran study in the United States also showed the decline of lung cancer risk since 1955 using the Cox-proportional hazard model [25]. However, the association between change in lung cancer risk and year of birth cohort did not adjusted for age effect and period effect. On the other hands, in recent years, the issue about the causal role of human papilloma virus (HPV) to the risk of some oropharyngeal cancer is being magnified [26]. Even though HPV has contributed some part of recent stable or increasing trend in incidence rate of some type of oropharyngeal cancer in western countries [27], the decline of incidence and mortality rate for oropharyngeal cancer over the past 20 years was greatly due to the decrease in smoking, which is the primary risk factor for these cancers [28]. Our findings showed that birth cohorts born in the 1920–1930s, however, had the highest mortality rates compared with successive cohorts. These individuals were already in early adulthood when manufactured cigarettes began to become widely available (after 1945) [2] and were probably more likely to have a somewhat higher lifetime exposure to cigarette smoking compared with those born in the 1950–1960s who would have experienced the tobacco control policy introduced in Korea in 1976 [29]. We hypothesized that changes in birth cohort would be similar between smoking-related cancers, if smoking was the major factor that affected the trends in mortality rates for these smoking-related cancers. Indeed, our finding showed that changes in mortality rate by birth cohort were similar between oropharyngeal, esophageal, laryngeal and lung cancer. Similarly, lung cancer mortality in Korean women seems to be highest among cohorts born in the 1920s [24,30].

In our study, the mortality rates of esophageal and laryngeal cancer decreased from 1996. Although it was difficult to establish smoking rates in Korea, the smoking rate seemed to reach its peak (79.3% among Korean men), in the 1980s and then to have leveled off [29]. The Korean tobacco control policy, which gives health warnings on cigarette packages, was introduced in 1976 [29]. In 1995, the National Health Promotion Act, which restricted smoking areas and cigarette advertising, was established [3]. The systematization and legislation of the anti-smoking movement resulted in the initiation and continuation of the smoking-related cancer mortality decline.

Although we could not show the change of trends in mortality rate for cardiovascular disease or chronic obstructive lung disease, it is obvious that many people also died from smoking related cardiovascular disease or respiratory disease in Korea. The nationwide representative study showed that about 35% of Korean men aged >45 years old with 20 pack-years of smoking had chronic obstructive pulmonary disease [31]. In addition, smoking accounts for about 45.1% of ischemic heart disease among young men aged <45 years old [32]. In this context, it is important to know the extent of reduction of smoking-related mortality, because it could be helpful to assess the effectiveness of tobacco control policy and enforce the future tobacco control policy. Our study suggested that reduction in cigarette smoking was associated with a decrease in the mortality of oropharyngeal, laryngeal, esophageal, and lung cancer, which account for a large proportion of deaths. Although current smoking rates have been decreasing among Korean men, smoking rates have not decreased among Korean women, especially among young women aged between 19 and 29 years [4]. While the Korean government has increased cigarette prices (in 2015), plain packaging for cigarettes has not been adopted. In addition to the decline in smoking related cancer, it should be considered that smokers could be died from other causes other than cancer—e.g., cardiovascular disease and chronic obstructive disease. Therefore, future study is needed to know the number and extent of death due to smoking and the burden of disease and cost of smoking. In addition, tobacco control policy should be more strongly implemented and supported to prevent smoking-related disease morbidity and mortality. Given that anti-smoking policies are effective in decreasing mortality from smoking-related cancers, our government should consider adopting plain packaging for cigarettes [33] and graphical health warning labels on cigarette packages [34].

Our findings have some limitations. Firstly, it is impossible to investigate a causal relationship or to calculate an attributable fraction, because our study was not an individual-based study. Secondly, the accuracy and completeness of the mortality data in Korea has gradually improved, but may have inaccuracies. Therefore, it could bias the estimates of either changes in mortality trends or birth cohort effects. Although smoking is the most important single factor which accounts for >50% of smoking-related cancer risk in our study, changes another risk factors for smoking-related cancer could be included in the cohort effects. Therefore, the interpretation of changes in birth cohort effects of smoking-related cancer mortality should be caution, because historical differences, history of infection epidemics, and changes of life style except smoking habit were involved in birth-cohort effects. In addition, it was impossible to analyze the age-period-cohort effects for incidence rate of smoking-related cancer, because the time period for cancer incidence was relatively short. Despite these limitations, our findings are useful to examine the secular trends in smoking-related cancer mortality. This study demonstrates that smoking-related cancer mortality follows similar birth cohort effects, suggesting that this reduction was mainly due to decreases in smoking rates.

## 5. Conclusions

In summary, oropharyngeal, esophageal, laryngeal, and lung cancer followed similar birth cohort effects, although the time points at which the mortality rate decreases began were slightly different slightly for each particular cancer. The similar cohort patterns for four types of smoking-related cancers suggest that the reduction in smoking rate led to a decrease in the cohort effect on smoking-related cancer mortality.

## Figures and Tables

**Table 1:** Baseline characteristics of overall and smoking-related cancer deaths during the study period (1984–2013) in Korean men.

Categories	Total Cancer Deaths *		Tumor Sites							
			Oropharynx		Esophageal		Larynx		Lung	
	N	%	N	%	N	%	N	%	N	%
**Total**	1,024,976	100.0	14,679	1.4	34,782	3.3	15,356	1.5	222,097	22.7
**Age group**										
<40 years	52,415	5.1	604	4.1	234	0.7	217	1.4	3214	1.4
40–49 years	99,598	9.7	1429	9.7	1707	4.9	764	5.0	10,632	4.8
50–59 years	214,102	20.9	3347	22.8	7764	22.3	3123	20.3	35,164	15.8
60–69 years	300,422	29.3	4619	31.5	12,970	37.3	5400	35.2	73,694	33.2
≥70 years	358,439	35.0	4680	31.9	12,107	34.8	5852	38.1	99,393	44.8
**Year of death**										
1984–1993	235,172	23.0	2212	15.1	8754	25.2	4512	29.4	34,586	15.6
1994–2003	351,795	34.3	5000	34.0	12,923	37.1	6582	42.9	77,197	34.7
2004–2013	438,009	42.7	7467	50.8	13,105	37.7	4262	27.7	110,314	49.7

* Total cancer deaths indicate cancer deaths for all sites combined (C00–C97). Cancer deaths are expressed as numbers and percentages by tumor sites. Data was obtained from Statistics Korea (Available from: http://kosis.kr/) [4].

## Supplementary Materials

The following are available online at www.mdpi.com/1660-4601/13/12/1208/s1. Table S1, Goodness of fit of age-period-cohort model assessment for smoking related cancer mortality in Korea.
