# Supplementary material for: Decreases in Smoking-Related Cancer Mortality Rates Are Associated with Birth Cohort Effects in Korean Men
Source: Int J Environ Res Public Health. 2016 Dec 5;13(12):1208. doi: 10.3390/ijerph13121208 (PMC5201349; doi:10.3390/ijerph13121208)
Supplement: Supplementary file 1 [file ijerph-13-01208-s001.pdf]

Supplementary Materials: Decreases in Smoking-Related Cancer Mortality Rates Are Associated with Birth Cohort Effects in Korean Men

Yonho Jee, Aesun Shin, Jong-Keun Lee and Chang-Mo Oh

Table S1. Goodness of fit of age-period-cohort model assessment for smoking related cancer mortality in Korea.

Model	Oropharyngeal Cancer				Laryngeal Cancer				Esophageal Cancer				Lung Cancer			
	df	Log-Likelihood	AIC	*p*-Value	*df*	Log-Likelihood	AIC	*p*-Value	*df*	Log-Likelihood	AIC	*p*-Value	*df*	Log-Likelihood	AIC	*p*-Value
Age	45	−579.9	21.8	<0.05	45	−2370.2	88.1	<0.05	45	−1991.0	74.1	<0.05	45	−8854.3	328.3	<0.05
Period	48	−6544.3	242.6	<0.05	48	−10,978.8	406.8	<0.05	48	−22,316.7	826.8	<0.05	48	−169,949.0	6294.6	<0.05
Cohort	40	−3255.1	121.1	<0.05	40	−1469.6	54.9	<0.05	40	−4714.1	175.1	<0.05	40	−61,567.3	2280.8	<0.05
Age + period	40	−334.5	12.9	<0.05	40	−786.9	29.7	<0.05	40	−1001.8	37.6	<0.05	40	−5728.2	212.7	<0.05
Age + cohort	32	−336.3	13.3	<0.05	32	−494.1	19.1	<0.05	32	−362.4	14.2	<0.05	32	−1326.0	49.9	<0.05
Period + cohort	35	−394.4	15.3	<0.05	35	−377.6	14.7	<0.05	35	−1188.7	44.7	<0.05	35	−2261.1	84.4	<0.05
Age + period + cohort (intrinsic estimator)	28	−242.9	10.0		28	−221.1	9.2		28	−239.6	9.8		28	−295.6	11.9	

df: degree of freedom; AIC: akaike information criterion.

© 2016 by the authors; licensee MDPI, Basel, Switzerland. This article is an open access article distributed under the terms and conditions of the Creative Commons by Attribution (CC-BY) license (http://creativecommons.org/licenses/by/4.0/).
